# Drivers, Barriers and Unmet Needs Affecting Feline Vaccination Compliance: Insights from a Global Survey of Cat Owners and Veterinarians

**DOI:** 10.3390/vetsci13070646

**Published:** 2026-06-30

**Authors:** Ericka L. Carroll, Nouran Nawar, Melissa Bourgeois

**Affiliations:** Merck Animal Health, 126 E. Lincoln Avenue, Rahway, NJ 07065, USA; ericka.carroll.vmd@gmail.com (E.L.C.); nouran.nawar@merck.com (N.N.)

**Keywords:** feline vaccination, vaccine compliance, vaccination barriers, client education, vaccine hesitancy, feline demographics

## Abstract

This online survey of 1330 veterinarians and 5071 cat owners across 14 countries aimed to identify drivers, barriers and unmet needs regarding feline vaccination compliance. Veterinarians and cat owners differed on the perceived value of vaccination; 16% of cat owners cited vaccination as a key contributor to feline health compared with 69% of veterinarians. Cat owners reported that 71% of their cats had seen a veterinarian within the previous year and 65% were previously vaccinated, aligning with veterinarian-reported compliance rates of 77% for core vaccines. Primary vaccination drivers for cat owners were the desire to maintain their cat’s health (62%) and a veterinarian’s recommendation (57%); veterinarians perceived vaccine reminders (71%) as the top driver. Owners who elected not to vaccinate their cats believed vaccines were unnecessary for healthy (27%) or indoor cats (27%). This aligned with veterinarian-reported barriers: lack of client understanding (53%), vaccine misinformation (51%) and antivaccine attitudes (51%). Most cat owners (58%) cited their veterinarian as their primary vaccine information source. However, only 23% of veterinarians report discussing the importance of vaccination with cat owners. Cat owners prioritized low side effects (88%), multi-antigen protection (86%) and >1 year of protection (86%; 74% want 3 years). Concerning price and compliance, 87% (61% yes, 26% maybe) would pay more for long-lasting protection, and 91% (66% yes, 25% maybe) of vaccinating owners would attend annual wellness visits even when vaccination is not due. By addressing educational gaps, communicating the role vaccines play in feline health, and understanding cat owner concerns and preferences, veterinarians have an opportunity to improve feline vaccination compliance.

## Simple Summary

Vaccination protects cats from diseases, yet many cats remain unvaccinated. We conducted online surveys of over 1300 veterinarians and 5000 cat owners from 14 countries, finding that veterinarians and cat owners have different vaccination views. Most veterinarians (69%) believe vaccination is key to keeping cats healthy, but only 16% of cat owners identified vaccination as a top contributor to cat health. About two-thirds of cats were vaccinated. Owners of vaccinated cats were motivated by a desire to keep cats healthy (62%) and follow their veterinarian’s recommendation (57%). Veterinarians agreed that their recommendation (71%) was a motivator. Interestingly, 71% of veterinarians believed veterinary reminders encouraged vaccination, but only 27% of cat owners mentioned reminders as a motivator. Cat owners’ decisions not to vaccinate were often based on false beliefs that vaccines were unnecessary for healthy (27%) or indoor (27%) cats. Veterinarians listed misinformation (51%) and owner lack of understanding (53%) as challenges to recommending vaccines. Most owners (58%) rely on their veterinarian for vaccine information, but fewer than one in four veterinarians discuss the importance of vaccination. Owners preferred vaccines with low side effects (88%), multi-antigen protection (86%) and >1 year of protection (86%). By improving veterinary communication about cat vaccination, more cats could be protected from preventable diseases.

## 1. Introduction

Feline vaccination is a critical component of veterinary care, offering individual protection against infectious diseases and contributing to community-wide herd immunity [1]. The World Small Animal Veterinary Association (WSAVA) designates feline parvovirus (FPV), feline herpesvirus-1 (FHV) and feline calicivirus (FCV) as core vaccines for all cats [2]. Where feline leukemia virus (FeLV) is endemic, FeLV vaccination is also considered core for cats <1 year of age and adult cats with outdoor access or residing with cats with outdoor access [2]. Rabies is also a core vaccine in endemic areas. Non-core vaccines for at-risk cats include feline immunodeficiency virus (FIV), *Chlamydia felis* and *Bordetella bronchiseptica* [2].

Despite these recommendations, a notable proportion of cats remain unvaccinated. In a 2019 study conducted in Germany, 77.9% of cats were appropriately vaccinated, and 5.4% had never been vaccinated [3]. For cats to be vaccinated, their owners must first seek veterinary care. According to 2024 surveys of cat owners in the United States (US), the United Kingdom (UK) and Ireland, just 57–62% of cats had seen a veterinarian within the preceding year [4,5,6]. In Argentina, fewer than 35% of owned pets are estimated to be vaccinated [7]. In Brazil, approximately 9% of cats receive annual veterinary visits and vaccines [7]. Data on vaccination rates in Asia are limited, but significant gaps in companion animal vaccination rates are recognized [8,9,10].

These statistics underscore the need for expanding care in feline patients. Measures such as the Cat Friendly Practice (CFP) program, a global initiative designed to enhance the care of cats by reducing stress during veterinary visits, are working to overcome barriers and increase veterinary care utilization among cat owners [11,12,13,14,15,16,17,18]. These measures positively impact care, with patients at CFP-accredited clinics receiving veterinary care more frequently than those cared for at non-CFP-accredited clinics [19].

This is the first multinational analysis to examine feline vaccination drivers and barriers from the perspectives of both cat owners and veterinarians. This study aims to discover global drivers and barriers to feline vaccination, from the perspectives of cat owners and veterinarians, and alignments and misalignments between these two groups. The study also aims to identify gaps and unmet needs that, if addressed, may improve feline vaccination compliance.

## 2. Materials and Methods

This quantitative study collected data through two online surveys, one for veterinarians and the other for cat owners, administered by a professional market research organization (Kynetec, St. Louis, MO, USA), adhering to global market research guidelines and codes of conduct. The study captured data from a representative sample of 1330 companion animal veterinarians and 5071 cat owners across 14 countries: the US, Canada, Japan, Brazil, Mexico, Australia, the UK, France, Germany, Italy, Poland, Belgium, the Netherlands and Denmark. The number of respondents from each country can be seen in Table 1 below.

Qualifying cat owner participants (qualified respondents) were 18+ years of age, owned 1–5 cats and were primary or shared decision makers regarding their cat’s healthcare. To reduce bias, participants affiliated with animal health companies or market research firms were excluded. The study targeted a representative sample of owner demographics, including geography, gender, age and household income. The survey took approximately 16 min to complete and was conducted from 16 May 2024 to 2 August 2024. Qualified respondents were asked about their cat’s vaccination status, followed by a series of questions assessing attitudes, behaviors, drivers and challenges related to vaccination. If a respondent had multiple cats, one cat was randomly selected as the focus for relevant questions throughout the survey. The cat owner survey is included in Supplemental Questionnaire S1.

Qualifying veterinarian participants (qualified respondents) were full-time veterinarians who spent at least 70% of their professional time treating companion animals, with at least 20% specifically on cats, had been practicing veterinary medicine for 2–40 years, were vaccine decision-makers at their practices and vaccinated feline patients. The study targeted a representative mix of participant demographics, such as gender and age, and practice demographics, such as location, size and ownership type (corporate versus independent). Participants were excluded if they were competitively employed or serving in an advisory capacity to animal health, market research and/or pharmaceutical companies. The survey was fielded from 29 May 2024 to 9 September 2024 and took approximately 29 min to complete. Qualified participants answered questions assessing their approach to feline vaccination, their perception of cat owners’ behavior regarding vaccination, and challenges related to feline vaccination. The veterinarian survey is included as Supplemental Questionnaire S2.

Responses were collected and analyzed in anonymized form. Open-ended free-text responses were coded and grouped by topic for descriptive summarization. The analysis was primarily descriptive and exploratory, with subgroup comparisons conducted to identify potential differences across designated groups. Binary outcomes were compared between groups using independent-samples *t*-tests on proportion data. Although z-tests are commonly used for comparisons of proportions, the independent-samples *t*-test was retained because it was the prespecified method in the analysis plan and was applied as a large-sample approximation for group-level percentages. Statistical significance was assessed at alpha = 0.10 (90% confidence level) for exploratory purposes. The statistical package used was WinCross Desktop Version 23. Because the study was intended to be descriptive and hypothesis-generating rather than confirmatory, results should be interpreted cautiously. No formal adjustment for multiple comparisons was applied.

## 3. Results

### 3.1. Cat Owner Demographics

Most survey respondents were female (56%). Just below half (47%) lived in urban areas, followed by suburban (30%) and rural (23%) areas, based on self-classification of the respondents. They ranged in age from 18 to 65+ years; the largest age demographic was 55+ years (34%). Most respondents were married or in a domestic partnership (64%) and lived in households of three or more people (54%). Most households (61%) owned other species of pets, notably dogs (47%).

### 3.2. Cat Demographics

Owners reported a total of 7762 cats, averaging 1.5 cats per household. Sixty-two percent of households were single-cat homes, 28% had two cats and 10% had 3+ cats. Most cats were mixed breed (63%); a smaller percentage (20%) were purebred. Seventeen percent of cat owners did not know their cat’s breed. Most cats (53%) were 1–6 years old, followed by 7–10 years (21%), 11+ years (17%) and <1 year old (9%). Owners reported that 41% of their cats live indoors only, while 3% live completely outdoors. Most cats live indoors but also spend time outside (56%). Cats were most frequently obtained from acquaintances (28%), found as a stray (22%) or adopted from an animal shelter (18%).

### 3.3. Veterinarian Demographics

Most respondents were female (68%). On average, they worked 41 h per week, spent 40% of their professional time treating cats and saw 47 cats per week. They had varying levels of clinical experience; the largest number of respondents had been in practice for 2–10 years (41%), followed by 11–20 years (34%), 21–30 years (19%) and 31–40 years (7%). Fifty-three percent worked as associate/employee veterinarians and 47% were practice owners/partners. The sample ensured representation of veterinarians employed by corporate veterinary practices, most notably in the UK and the US, and by independent practices.

### 3.4. Contributors to Feline Health and Wellness

**Figure 1:**
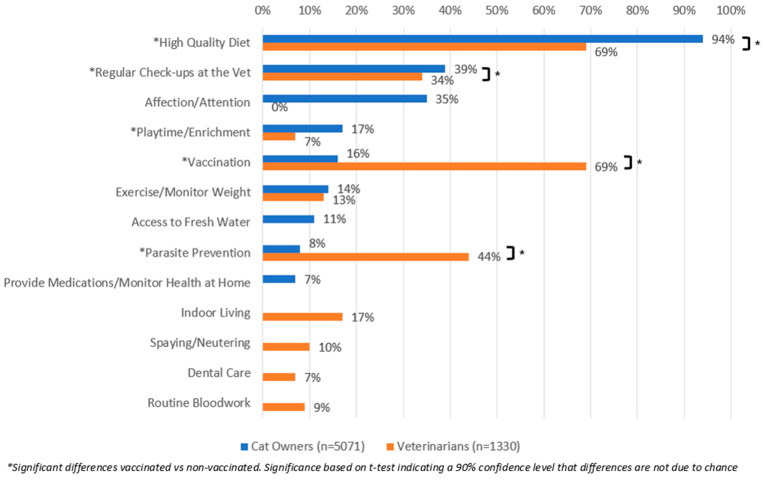
Top contributors to feline health as identified by cat owners and veterinarians.

When asked to unaidedly identify three key contributors to maintaining feline health, the most frequent response among cat owners was a high-quality diet (94%), with vaccination only cited by 16% of respondents. Veterinarians were also unaidedly asked to identify the three most important actions a cat owner could take to maintain their cat’s health. Feeding a high-quality diet (69%) and vaccination (69%) tied for the top response. Key health contributors reported by cat owners and veterinarians when asked unaidedly are presented in Figure 1.

In a relative ranking exercise, in which respondents identified the most and least important factors for maintaining feline health, vaccination was ranked sixth, following diet, illness/injury monitoring, parasite prevention, comfortable environment and annual veterinary examinations.

### 3.5. Vaccine Compliance

Based on cat owner responses, 71% of cats had seen a veterinarian in the last 12 months and 65% were previously vaccinated. If a client had multiple cats, one cat was randomly selected for a series of relevant questions. Of those cats, 68% had been vaccinated in the last 12 months, while 7% had not received a vaccine in over 3 years.

When asked what percentage of cat owners comply with vaccination recommendations, veterinarians reported a compliance rate of 77% for core vaccines and 49% for non-core vaccines.

### 3.6. Drivers of Vaccination

Owners of vaccinated cats reported the leading factors influencing their decision to vaccinate were a desire to maintain their cat’s health (62%) and their veterinarian’s recommendation (57%). Other major drivers included the cat’s lifestyle, and vaccine reminders. Of the 27% of cat owners who reported vaccination reminders influenced their decision, they reported a preference for email (42%) or text message (38%) reminders over phone calls (10%) and postcards (8%).

**Figure 2:**
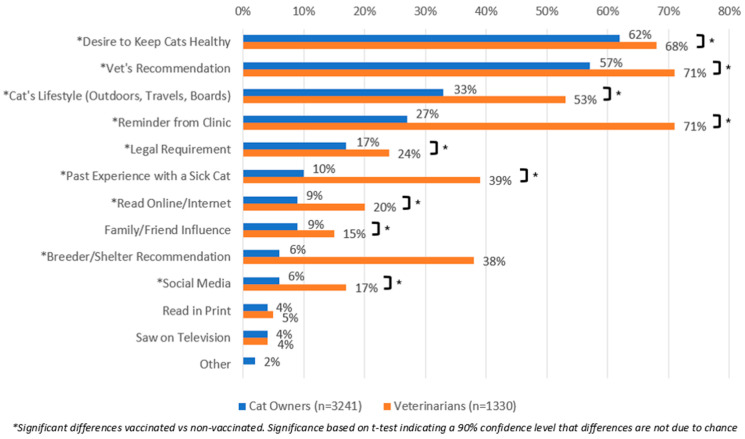
Drivers to vaccinate from cat owners of vaccinated cats and veterinarian perceptions.

Veterinarians reported vaccine reminders (71%) and their recommendations (71%) as the top perceived drivers of vaccination. Other key drivers include the client’s desire to keep the cat healthy and the cat’s lifestyle. Vaccination drivers reported by cat owners and veterinarians are presented in Figure 2.

### 3.7. Indoor vs. Outdoor Cats

Indoor and outdoor cats were classified according to lifestyle as “lives indoor and never goes outside” (n = 1965), “lives indoors and rarely goes outside” (n = 1147), “lives indoors and routinely goes outside” (n = 1814), “lives exclusively outdoors” (n = 145). Indoor and outdoor cats share the same foundational needs—good diet and regular monitoring for signs of illness or injury—but their preventive-care priorities and veterinary behaviors differ. Within the last 12 months, indoor-only (72%), mostly-indoor cats (77%), and cats that live indoors and routinely go outside (77%) were more likely to have visited the veterinarian than exclusively outdoor cats (43%).

Across all groups, wellness and vaccination are the primary reasons owners take cats to the vet (67%). Owners who vaccinate are most commonly motivated by veterinary recommendations and a desire to keep their cats healthy across all indoor and outdoor cat groups; however, lifestyle (i.e., an outdoor routine) also strongly influences vaccination decisions for owners of outdoor only cats (52%) and cats that live indoors and routinely go outside (54%) vs. indoor only cats (1%) and cats that live indoors and rarely go outside (24%). Among those who do not vaccinate, common reasons include believing the cat is healthy for all groups (26–39%) and, for indoor-only cats, that limited contact with other animals reduces the need for vaccines (23%); cost is a concern for all owners but slightly less so for indoor-only households. Lower-cost options, discounts or promotions are the top motivators that could persuade non-vaccinators to seek vaccination, while specific incentives appeal to subgroups—outdoor-only cat owners respond particularly well to mobile clinics (51%), payment plans (49%), and guidance on transporting cats (38%). Finally, getting a cat into a travel carrier is the single biggest barrier to attending vet appointments for all owners, and is especially problematic for outdoor-only cat owners, who are also more likely to struggle with finding their cat in time to leave for an appointment.

### 3.8. Barriers to Vaccination

Among owners of vaccinated cats, the most frequently reported barriers to care included feline handling, stress, and cost. However, 16% of respondents noted that they do not face any challenges. Results are presented in Table 2.

Owners of vaccinated cats were asked unaidedly how their veterinary practice could help overcome these barriers. Top responses included home visits (13%), less stressful/cat-friendly environment (10%), discounts (8%), flexible scheduling (3%) and pre-visit calming medications (3%). Twenty-eight percent of respondents noted no need for help and 10% were unsure. When prompted with a list of measures that may influence their decision to continue vaccinations, cat owners cited lower costs/discounts (58%), convenient appointment times (57%) and recommendations from the veterinarian (56%) as top influencers.

**Figure 3:**
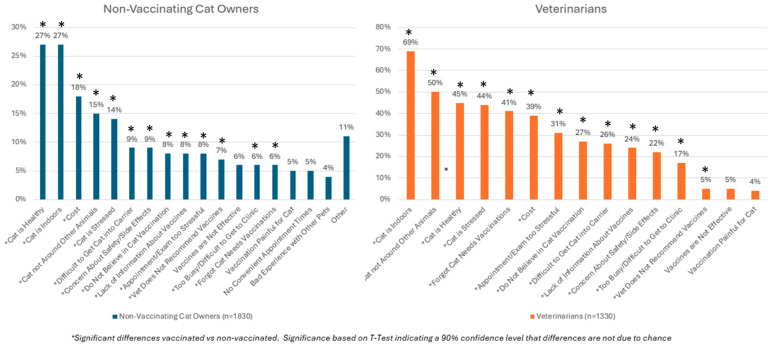
Drivers for a cat owner NOT to vaccinate and veterinarians’ perceptions-results from non-vaccinating cat owners and veterinarians.

Among owners of unvaccinated cats, top drivers of their decision not to vaccinate were that the cat was healthy (27%) or lived indoors (27%). When veterinarians were asked why they believed cat owners would decline vaccination, they reported misconceptions regarding the need for vaccination of indoor cats (69%), cats that do not encounter other animals (50%) and healthy cats (45%) as primary reasons. Results are presented in Figure 3.

Owners of unvaccinated cats were unaidedly asked how a veterinary practice could encourage vaccination. Top responses included reduced costs (26%) and information/education on vaccination (24%). When prompted with a list of potential measures that may motivate them to vaccinate their cat, reduced costs (54%), convenient appointment times (39%) and information on vaccine safety (38%) were selected most frequently.

Veterinarians reported the most common barriers in recommending vaccinations during veterinary visits included clients not understanding the importance of vaccines (53%), vaccine misinformation (51%) and anti-vaccination attitudes (51%). Results are presented in Table 3.

### 3.9. Promoting Vaccination: Client Education and Other Strategies

**Figure 4:**
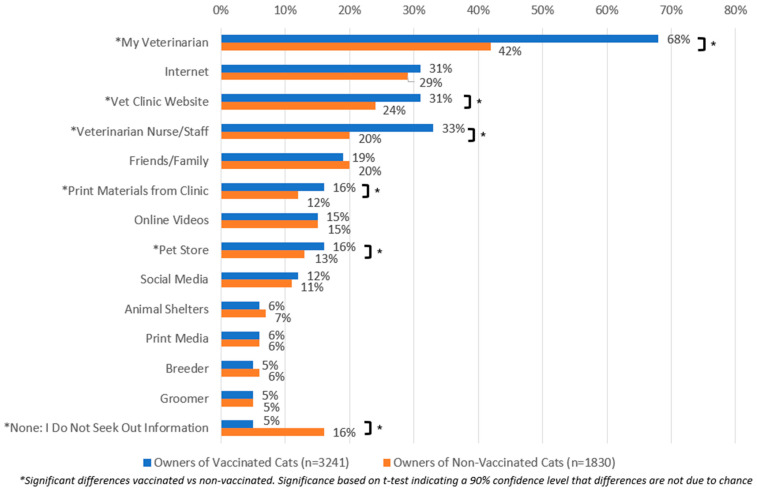
Information sources used to learn about vaccines by owners vaccinating and not vaccinating their cats.

Fifty-eight percent of cat owners cited their veterinarian as their primary source of vaccine information and 28% cited veterinary nurses/staff. Thirty percent indicated that they use the internet as an information source; among these respondents, their veterinary practice’s website (53%) and practice emails (40%) were top resources, followed by online videos (24%), vaccine manufacturer’s website (21%), pet blogs (15%), social media (14%), and other (4%). Sixteen percent of owners reported using printed materials from the practice as an information source. While owners of both vaccinated and unvaccinated cats agreed on top sources of information, owners of vaccinated cats were more likely to cite veterinarians (68% vs. 42%), practice websites (31% vs. 24%) and veterinary teams (33% vs. 20%) than owners of unvaccinated cats. These results are presented in Figure 4.

Veterinarians were asked how they encourage feline vaccination in their practice. Seventy-eight percent reported sending vaccination reminders and most (73%) reported this to be effective. Other methods that were commonly used and viewed as effective included checking the records of all household pets when any pet is presented for a veterinary visit, and offering resources for stress-free feline visits. Results are presented in Table 4.

The 23% of veterinarians who reported sharing educational materials regarding the importance of feline vaccination were asked what materials were used most often. In-practice printed materials (70%), social media posts (45%) and emails (33%) were cited as the top materials used.

### 3.10. Promoting Vaccination: Client Preferences

When owners were asked about vaccine preferences, prioritization was placed on low side effects (88%), multi-antigen protection (86%) and >1 year of protection (86%; 74% want 3 years). Concerning price and compliance, 87% (61% yes, 26% maybe) would pay more for long-lasting protection, and 91% (66% yes, 25% maybe) of vaccinating owners would attend annual wellness visits even when vaccination is not due.

## 4. Discussion

To the authors’ knowledge, this is the first multinational analysis to examine feline vaccination drivers and barriers from the perspectives of both cat owners and veterinarians. The study’s objective was to identify gaps and unmet needs that, if addressed, could improve vaccination compliance. Prior surveys have largely focused on cat owners in the UK and US, reporting the proportion of cats vaccinated or whether vaccination motivated a veterinary visit (3–6), with vaccination rates ranging from 70% to 77%. Our findings are broadly comparable, with owners reporting that 65% of cats had been vaccinated. However, data outside Europe and North America remain limited; for example, regional WSAVA materials provide only estimates for Latin America (7). Importantly, previous surveys did not systematically assess reasons for vaccination compliance or non-compliance—knowledge that is critical for understanding owner decision-making and for designing effective interventions. One study of German cat owners explored motivations and barriers (3), but its finding that regulatory requirements were the strongest driver of vaccination may not generalize across countries with different legal frameworks. By incorporating insights from both veterinarians and cat owners across 14 countries, the present study adds essential, globally relevant evidence to deepen understanding of feline vaccination compliance and to inform strategies for improvement.

This study reveals substantive divergences between veterinarians and cat owners regarding the perceived role of vaccination in feline health and wellbeing. Veterinarians rated vaccination on par with diet as a key health-maintenance measure, whereas owners strongly prioritized diet and assigned considerably less importance to vaccination. Despite this attitudinal gap, reported veterinary visitation within the preceding year (71%) exceeded recent estimates from the US, UK, and Ireland (57–60%), suggesting potential progress in feline access to care. However, differences in sampling, survey phrasing, demographics, and owner reporting cannot be excluded as alternative explanations. Taken together, these findings indicate both an encouraging trend in care-seeking and a persistent opportunity to enhance vaccine understanding and uptake among cat owners.

Vaccination coverage remains suboptimal relative to guideline recommendations. Although WSAVA guidelines advocate core vaccines for all cats, veterinarians reported a core vaccine compliance rate of 77%, and only 65% of owners indicated their cat had ever been vaccinated. The drivers of vaccination among participating owners were primarily a desire to maintain their cat’s health and explicit recommendations from veterinarians, and veterinarians likewise identified their recommendation as the predominant influence on owner behavior. Conversely, owners of unvaccinated cats most frequently cited misconceptions—such as believing vaccines are unnecessary for healthy, indoor, or low-contact cats—over cost considerations. This is clearly verified in the sub-group differences between indoor-only cats and cats with outdoor access or outdoor only cats. Veterinarians recognized similar barriers, including limited client appreciation of vaccine value and misunderstandings about indications and benefits. These patterns underscore the central role of veterinarians in shaping vaccine decisions and the need for targeted education to address prevalent misconceptions.

Information channels present a practical lever for improvement. While veterinarians were the most important source of vaccine information for owners of both vaccinated and unvaccinated cats, relatively few practices reported sharing educational materials on feline vaccination. Among those that did, print predominated, despite owners’ stated preference for digital resources—particularly practice websites and emails. Practices also reported employing operational strategies such as reminders, universal vaccination status checks, opportunistic scheduling during other visits, and wellness plans. Although most veterinarians perceived reminders as effective, a minority of owners identified reminders as a key driver of vaccination decisions, suggesting a need to refine content, timing, and modality to better align with owner preferences.

These findings highlight actionable opportunities for veterinary practices to strengthen vaccination uptake. First, vaccine-focused client education should directly address common misconceptions, emphasizing that core vaccines are indicated for all cats, including healthy, indoor, and low-contact cats, in accordance with established guidelines. Clear, concise messaging and FAQ-style materials on safety, necessity, and scheduling may improve comprehension and confidence. Second, aligning communication channels with owner preferences is essential: expanding digital-first education via practice websites and email—complemented by social media where appropriate—can increase reach and engagement, with mobile-friendly formats and follow-up links after visits. Third, consistent, explicit recommendations during routine interactions, supported by communications training and motivational interviewing techniques, may enhance receptivity and reduce hesitancy. Fourth, operational strategies such as universal vaccination status checks at every visit, point-of-care scheduling of vaccine appointments, tailored reminders that combine educational content with personalized prompts, and wellness plans that bundle preventive care can reduce friction and improve compliance. Finally, maintaining momentum in access to care through cat-friendly handling, practical transport guidance, and streamlined appointment pathways can help sustain annual visitation and create more opportunities to vaccinate.

This study has several limitations. The survey encompassed 14 countries and may not fully reflect attitudes and experiences in other regions. The owner sample was stratified by vaccination status, introducing potential selection bias. Vaccination status and compliance were self-reported, without verification via medical records, and responses may have been influenced by social desirability. The higher reported rate of veterinary visitation versus prior estimates could reflect genuine changes in utilization or differences arising from sampling methods, survey wording, demographics, or reporting behavior.

Future research should prioritize validation of vaccination status and compliance through medical record review to corroborate self-reports. Randomized evaluations of reminder strategies and multi-channel education—testing message framing, timing, and delivery modalities (email, SMS, website, social media, print)—are warranted to identify best practices that effectively shift behavior. Targeted educational interventions addressing specific misconceptions (e.g., vaccination needs for healthy, indoor, or low-contact cats) should be assessed for impact on knowledge, attitudes, and uptake. Pragmatic trials of practice-level interventions—such as universal status checks, point-of-care scheduling, and wellness plans—could quantify their effects on compliance and inform scalable implementation. Finally, expanding the geographic scope and including diverse populations will enhance generalizability and elucidate cultural and regional differences in vaccine attitudes, information preferences, and care-seeking behaviors.

## 5. Conclusions

There is an opportunity to improve feline vaccine compliance globally. This study identifies gaps that, if addressed, could improve feline vaccination rates. Although veterinarians recognize vaccination as essential to feline health, many cat owners undervalue its importance, often due to misconceptions about vaccine necessity. While cat owners report relying heavily on veterinarians for vaccine information, only about one in four veterinarians report sharing educational materials on the importance of vaccination. This gap underscores the need and opportunity for veterinarians to influence vaccination decisions through client education and communication.

This study also highlights several effective strategies utilized by veterinary practices to promote vaccination. Future efforts should focus on identifying best practices and fostering knowledge exchange among veterinary practices to increase vaccination rates and better protect more cats globally with vaccination.

## Figures and Tables

**Table 1:** Number of Respondents by Country and Respondent Type.

	Veterinarians	Owners
United States	200	500
Canda	103	400
UK	101	400
France	100	400
Germany	100	400
Italy	100	400
Poland	100	400
Belgium	50	250
Netherlands	50	250
Denmark	25	50
Brazil	100	400
Mexico	101	400
Japan	100	419
Australia	100	402

**Table 2:** Challenges reported by cat owners vaccinating their cats.

Challenge with Vaccination	Frequency Reported
Getting the cat into the travel carrier	36%
The stress during the examination (cat is crying, yowling, hissing, growling, and/or trying to bite/scratch the employees)	34%
The cost of the veterinary service and/or products	32%
Travelling to the veterinarian with the cat (cat is crying, yowling, vomiting, urinating, defecating, scared)	28%
Stress of encountering other animals at the clinic	22%
Obtaining an appointment at a convenient time for me	17%
Cannot find the cat when it is time to leave for the appointment (cat is hiding or is outside and cannot be found)	13%
Other	1%
I do not face any challenges when taking my cat to the vet	16%

**Table 3:** Challenges reported by veterinarians when recommending vaccines to cat owners.

Reported Barrier	Frequency Reported
Clients’ poor understanding of the importance of vaccination	53%
Addressing misinformation the cat owner has heard (from internet, breeder, etc.)	51%
Clients who are opposed to vaccines in general/anti-vaccination	51%
Client’s lack of interest in learning about vaccinations for their cats	40%
Discussing cost/navigating cost concerns expressed by cat owner	39%
Lack of client compliance with vaccine recommendations	39%
Not enough time during patient visit to discuss vaccination	17%
Clients’ lack of trust in vet recommendations	15%
Not enough consistency among all clinic teams in vaccine communication	10%
Nothing/no challenges or difficulties	5%

**Table 4:** Methods veterinary practices use to encourage feline vaccination and reported effectiveness.

Method Used by Clinic to Encourage Cat Vaccination Compliance	% of Veterinary Practices Using	Method Used by Clinic to Encourage Cat Vaccination Compliance
Vaccination reminders	78%	73%
Check vaccination records of all pets in the households during any pet’s visit	46%	62%
Offer therapies to help reduce appointment stress (pheromones, sedatives)	40%	49%
Forward book vaccine appointment during clinic visits	39%	60%
Offer a cat-friendly clinic experience	38%	56%
Use online booking for vaccine appointments	37%	48%
Use online booking for vaccine appointments	37%	48%
Educate owners on carrier use and transporting the cat	31%	38%
Posts about feline vaccination on clinic’s social media channels	25%	26%
Offer wellness and/or health plan	24%	64%
Share educational materials on importance of vaccination for cats	23%	27%

## Data Availability

The original contributions presented in this study are included in the article/Supplementary Materials. Further inquiries can be directed to the corresponding author.

## Supplementary Materials

The following supporting information can be downloaded at https://www.mdpi.com/article/10.3390/vetsci13070646/s1, Supplemental Questionnaire S1: Cat Owner Survey; Supplemental Questionnaire S2: Veterinarian Survey.

## Abbreviations

The following abbreviations are used in this manuscript:

WSAVA	World Small Animal Veterinary Association
FPV	Feline parvovirus
FHV	Feline herpesvirus-1
FCV	Feline calicivirus
FeLV	Feline leukemia
FIV	Feline immunodeficiency virus
US	United States
UK	United Kingdom
CFP	Cat Friendly Practice
