# Supplementary material for: Drivers, Barriers and Unmet Needs Affecting Feline Vaccination Compliance: Insights from a Global Survey of Cat Owners and Veterinarians
Source: Vet Sci. 2026 Jun 30;13(7):646. doi: 10.3390/vetsci13070646 (PMC13431493; doi:10.3390/vetsci13070646)
Supplement: Supplementary file 1 [file vetsci-13-00646-s001.zip › Global Feline Vaccines_VETS_Survey.docx]

**Supplemental Questionnaire 2- Veterinarian Survey**

- TARGET RESPONDENTS / RESPONDENT QUALIFICATIONS:

A random representative sample of veterinarians meeting the following criteria:

- Full-time practicing owner, partner or associate/employed veterinarian (GPs)
- At least 70% of professional time spent seeing and treating dogs and/or cats and at least 20% of their professional time seeing and treating cats
- Have been practicing at least 2 but not more than 40 years
- Decision maker or involved in the decision-making process regarding the vaccines stocked and recommended in the practice
- Must use vaccines for their feline patients
- Is not competitively employed or serving in an advisory capacity to an animal health, market research, and/or pharmaceutical companies.
- Representative mix of demographic and firmographic practice characteristics (region, practice size, gender, age, etc.)
- METHODOLOGY: Online survey
- BUDGETED SURVEY LENGTH: 20-minutes

SURVEY BEGINS HERE FOR RESPONDENTS. ANY TEXT FOUND IN PURPLE COLOR, INSIDE BRACKETS [ ] OR IN BOLD BLUE FONT IS FOR INTERNAL USE ONLY AND WILL NOT BE SEEN BY RESPONDENTS.

WELCOME SCREEN

Thank you for your interest in our study. Your opinions are very important to us!

This is a country-wide study designed to represent veterinarians from varying situations. For this reason, the first section of this survey consists of a few classification questions.

If you fit into one of the groups of veterinarians that are needed for this research, you will be given the option to participate in this survey. If you do not fit into one of the groups of veterinarians currently needed to complete the survey, we want you to know that we appreciate your interest in the study and hope that you will be able to participate in future research.

It will only take a few minutes to determine if you fit into one of the groups needed for this study.

In accordance with standard market research practices your identity will remain confidential. Your responses will not be linked with your name and your information will be used for research purposes only.

When you are ready to begin, please click "Next".

INTEGRITY QUESTIONS

Honest answers are important to the integrity of the research process. As such, please take the time necessary to provide thorough and thoughtful answers to this survey. If you attempt to complete the survey in an unreasonable amount of time or answer in nonsensical ways, you may be disqualified.

Before you begin, please check the box indicating agreement with our survey integrity policy.

- I agree to carefully read and respond as accurately as possible to all questions within this survey.

[PROGRAMMER: RESPONDENT MUST CHECK THE BOX BEFORE BEING ALLOWED TO MOVE ON TO THE SURVEY]

SCREENING QUESTIONS

OBJECTIVE: Screening criteria – Section S: S0, S10, S20, S30, S40, S50, S60, S70, S80, S90, S95, S100, S105, S110

S0. Are you licensed to practice veterinary medicine?

| Yes | 1 | [CONTINUE] |
| --- | --- | --- |
| No | 2 | [TERMINATE] |

S10. In what state/region/province is your practice located?

[SHOW DROP DOWN LIST OF APPROPRIATE STATES/REGION/PROVINCE BASED ON COUNTRY. EXCEL SHEET PROVIDED TO PROGRAMMING]

S20. What is your gender?

| Female | 1 |
| --- | --- |
| Male | 2 |
| Non-binary/third gender | 3 |
| Prefer to self-describe:_________________ | 4 |
| Prefer not to say | 5 |

S30. How many hours do you work in a typical week?

| Hours worked in a typical work week | **____** | [TERMINATE IF < 30 HRS; RANGE 0-999] |
| --- | --- | --- |

S40. Which of the following BEST describes your position…?

[SINGLE PUNCH]

| Veterinary practice owner or partner | 1 | [CONTINUE] |
| --- | --- | --- |
| Associate or employed veterinarian | 2 | [CONTINUE] |
| Veterinary nurse | 3 | [TERMINATE] |
| Veterinary technician | 4 | [TERMINATE] |
| Other clinic staff member | 98 | [TERMINATE] |

S50. How many years have you been practicing veterinary medicine?

| Years practicing veterinary medicine | ____ | [TERMINATE IF <2 or >40; RANGE 0-99] |
| --- | --- | --- |

S60. What percent of your professional time is spent seeing and treating…?

| Dogs | 1 | ____% |
| --- | --- | --- |
| Cats | 2 | ____% |
| Rabbits | 3 | ____% |
| Other species | 4 | ____% |
| **TOTAL** |  | [100%] |

[TERMINATE IF SUM OF CATS+DOGS <70%; CATS MUST BE AT LEAST 20%]

S70. In a typical week, approximately, how many of each of the following do you see?

| 1. Number of **Dogs** in a typical week [HIDE IF S60_DOGS=0] | ____ |
| --- | --- |
| 1. Number of **Cats** in a typical week | ____ |
| 1. Number of **Rabbits** in a typical week [HIDE IF S60_RABBITS=0] | ____ |

S80. In the past 12 months, thinking of all your patients that you saw, approximately what percent were vaccinated? Please answer separately for each species shown below.

| 1. % of **Dogs** vaccinated [HIDE IF S60_DOGS=0] | ____% |
| --- | --- |
| 1. % of **Cats** vaccinated | ____% |
| 1. % of **Rabbits** vaccinated **[HIDE F S60_3 (RABBITS)=0]** | ____% |

[TERMINATE IF CATS <10]

S90. And, would you **primarily** categorize yourself as…?

[SINGLE PUNCH]

| A general practitioner | 1 | [CONTINUE] |
| --- | --- | --- |
| A specialist that, for example, has a diploma, board-certification, or certificate in cardiology, neurology, oncology, orthopaedics, dermatology or some other specialty | 2 | [TERMINATE] |
| An emergency veterinarian that primarily provides emergency services | 3 | [TERMINATE] |
| A mobile or house call ONLY veterinarian | 4 | [TERMINATE] |
| A veterinarian that predominantly specializes in/predominantly practices alternative medicine | 5 | [TERMINATE] |

S95. Which of the following do you **predominantly** work for?

[RANDOMIZE LIST. SINGLE PUNCH]

| Independently owned, private practice | 1 | [CONTINUE] |
| --- | --- | --- |
| Corporate owned practice | 2 | [US & AU: MAX 30% OF COMPLETES  UK: MAX 50% OF COMPLETES  ALL OTHERS: MAX 15% OF COMPLETES] |
| Spay/Neuter or vaccine clinic [DO NOT SHOW IN PL] | 3 | [TERMINATE] |
| Animal shelter / rescue organization | 4 | [TERMINATE] |
| Other | 5 | [TERMINATE] |

S100. Do you currently work in or are you employed by any of the following types of businesses or professions? Select all that apply.

[RANDOMIZE LIST. MULTI-PUNCH]

| Pharmaceutical company | 1 | [TERMINATE] |
| --- | --- | --- |
| Military | 2 | [TERMINATE] |
| Manufacturing, distribution, sales or research of health care products | 3 | [TERMINATE] |
| A university **(full-time)** | 4 | [TERMINATE] |
| A university **(PART-TIME)** | 5 | [CONTINUE] |
| Governmental agency/entity [IN US, SHOW: including the FDA, USDA, EPA, and other agencies] | 6 | [TERMINATE] |
| None of the above [MUTUALLY EXCLUSIVE] | 99 | [CONTINUE] |

[TERM RESPONDENTS IF THEY SELECT ANY OF THE TERMINATION POINTS]

S105. Are you currently advising or providing consulting services to any animal health companies for a fee or engaged in studies sponsored by a pharmaceutical or health care company?

| 1 | Yes [TERMINATE] |
| --- | --- |
| 2 | No [CONTINUE] |

S110. What role do you play in making decisions related to the vaccines **carried and/or used** in your practice? Would you say that you…?

[SINGLE PUNCH]

| Are the sole decision maker | 1 | [CONTINUE] |
| --- | --- | --- |
| Share in the decision-making responsibility, or have input into the decision-making process | 2 | [CONTINUE] |
| Have little to no role in decisions regarding vaccines carried and/or used in the practice | 3 | [TERMINATE] |

TERMINATION TEXT

[IF RESPONDENT SCREENS OUT, SHOW:]

Unfortunately, you do not fit into one of the groups of veterinarians that we are currently seeking to take part in this study. However, your opinions are important to us, and we hope you will be able to participate with us in another future study. Thank you again and have a good day!

REPORTING OF ADVERSE EVENTS

PLACEHOLDER FOR UPDATED AE STATEMENT FROM MSD

MAIN QUESTIONNAIRE

SECTION A: VACCINE PROTOCOLS

OBJECTIVE – Understand current approach towards vaccinating cats

- - - What cat owners can do to keep cats healthy – QA1
    - Current protocols​ – QA2, QA3, QA3a, QA4, QA5
    - Frequency of and triggers for protocol review – QA6, QA7
    - Information sources used most often by vets for vaccine products – QA8

For today, please think only about your **CAT** patients.

QA1. When you think about your cat owner clients, in your opinion what are the top 3 things an owner can do to keep their cat healthy? *Please use a separate line for each response.*

[PRG: SHOW 3 TEXT BOXES]

Next, we would like to ask you a few questions that will help us understand more about the administration and use of vaccines for cats.

QA2. For your practice, which of the following vaccinations do you consider core CAT vaccinations, and which do you consider non-core?

By CORE, we mean those vaccines that all cats should receive regardless of their geography or lifestyle.

NON-CORE are those vaccines that are offered based on the risks associated with the lifestyle of the cat or where they live.

PGR: RANDOMIZE LIST

| **Vaccine** | | **Core** | **Non-Core** |
| --- | --- | --- | --- |
| 1 | Feline rhinotracheitis/herpesvirus (FVR/FHV-1) | ○ | ○ |
| 2 | Feline calicivirus (FCV) | **○** | **○** |
| 3 | Feline parvovirus (FPV) | **○** | **○** |
| 4 | Feline leukemia (FeLV) virus | **○** | **○** |
| 5 | Feline chlamydiosis / Chlamydia (C. *felis*) | **○** | **○** |
| 6 | Rabies | **○** | **○** |
| 7 | Bordetella bronchiseptica (Bb) [ONLY SHOW IN US AND NETHERLANDS] | **○** | **○** |

QA3. Of the **adult CATs** you personally see, approximately what percent are routinely vaccinated against each of the following diseases?

|  | VACCINE | **PERCENT OF CATS**  [RECORD PERCENT. ACCEPTED RANGE 0-100.] |
| --- | --- | --- |
| 1 | Feline rhinotracheitis/herpesvirus (FVR/FHV-1) | **____%** |
| 2 | Feline calicivirus (FCV) | **____%** |
| 3 | Feline parvovirus (FPV) | **____%** |
| 4 | Feline leukemia (FeLV) virus | **____%** |
| 5 | Feline chlamydiosis / Chlamydia (C. *felis*) | **____%** |
| 6 | Rabies | **____%** |
| 7 | Bordetella bronchiseptica (Bb) [ONLY SHOW IN US AND NETHERLANDS] | **____%** |

FOR ANY VACCINES THAT =0, FLAG AS ‘DO NOT USE’

PGR: SKIP QA3a IF NO NON-CORE VACCINES ARE USED [ALL VACCINES=CORE IN QA2 OR ALL NON-CORE VACCINES=0 IN QA3]

QA3a. Base=vaccines used and selected as non-core

You indicated that you consider each of the following as a NON-CORE vaccine for your cat patients.

From the list below please tell us for which type of cat / cat lifestyle you WOULD recommend the vaccine. Select all that apply in each column shown.

|  | [RANDOMIZE LIST] | [INSERT VACCINES SELECTED AS NON-CORE IN QA2 AND >0 IN QA3, 1 PER COLUMN]  **Recommended for…** |
| --- | --- | --- |
| 1 | Strictly indoor cats |  |
| 2 | Mostly indoor cats (rarely go outside) |  |
| 3 | Indoor / outdoor cats (routinely go outside) |  |
| 4 | Strictly outdoor cats |  |
| 5 | Cats that travel |  |
| 6 | Cats that will be boarded (in a cattery / kennel) |  |
| 7 | Single cat households |  |
| 8 | Multi cat households |  |
| 9 | Households that also have a dog(s) |  |
| 10 | Owners requesting the vaccine |  |
| 11 | Cats that go to cat shows |  |
| 12 | Kittens / cats under 1 year old |  |
| 91 | Other, please specify:__________________ |  |

QA4. Base=vaccines used

For each of the following diseases, please indicate your typical frequency of vaccination for **adult CATs** (with average exposure). Please make one selection per row.

|  | [RANDOMIZE ORDER IN WHICH LIST IS PRESENTED.] | **Every 6 Months** | **Every Year** | **Every 2 Years** | **Every 3 Years** | **Other frequency (please specify)** |
| --- | --- | --- | --- | --- | --- | --- |
|  | INSERT QA3 ROWS >0. INCLUDES CORE & NON-CORE. | 1 | 2 | 3 | 4 | 9 |

QA5. When developing your current vaccination protocol for cat patients, what factors were **most influential**? Select up to 5 factors.

[RANDOMIZE LIST, BUT KEEP ‘OTHER’ AND ‘NONE’ LAST]

| 1 | Guidelines published by industry associations (e.g., VGG, WSAVA, AAHA, etc.) |
| --- | --- |
| 2 | Knowledge from university / training |
| 3 | Manufacturer’s recommendation / data sheet |
| 4 | Protocol/opinion of veterinary colleagues |
| 5 | Protocol/opinion of key opinion leaders |
| 6 | Research/studies/literature from trusted industry sources |
| 7 | Risk of infection/disease in local area |
| 8 | Opinions/preferences of cat owner clients |
| 9 | Protocol/opinion of practice owner(s)/management |
| 91 | Other, please specify:__________________ |
| 99 | None [EXCLUSIVE] |

QA6. Approximately how often do you review your current vaccine protocols for your **CAT** patients to consider if any updates need to be made?

| 1 | Once per year |
| --- | --- |
| 2 | Once every 2-3 years |
| 3 | Once every 4-5 years |
| 4 | Once every 6+ years |
| 5 | Other, please specify:_____________ |

QA7. What are the **most important** factors that prompt / influence your decision to review or consider updating your **CAT** vaccine protocols? Select up to 5 factors.

[RANDOMIZE LIST, BUT KEEP ‘OTHER’ AND ‘NONE’ LAST]

| 1 | Scheduled review / when it is time for my planned review [ANCHOR FIRST] |
| --- | --- |
| 2 | Information at veterinary conferences / CE classes |
| 3 | Sales rep visits / training sessions / presentations |
| 4 | Information in industry journals/publications |
| 5 | Updated/revised guidelines from industry associations (WSAVA, AAHA, etc.) |
| 6 | New vaccine technologies introduced |
| 7 | New vaccine brand introduced (not new technology) |
| 8 | Client feedback |
| 9 | Recommendations from other veterinarians |
| 10 | Recommendations from key opinion leaders in the industry |
| 11 | Changes in risk level for a disease in my area (i.e., more cases being seen) |
| 91 | Other, please specify:_____________ [ANCHOR LAST] |

QA8. Which of the following sources do you use most frequently to get information about CAT vaccine products? Please answer separately for vaccines you are currently using and for new vaccine products not available in the past. Select up to 5 in each column.

| [RANDOMIZE LIST, BUT KEEP ‘OTHER’ AND ‘NONE’ LAST] | | New vaccine products not available in the past | Vaccines you currently use |
| --- | --- | --- | --- |
| 1 | Other veterinarians | ○ | ○ |
| 2 | Industry journals/publications | **○** | **○** |
| 3 | In-clinic visits with manufacturer sales reps | **○** | **○** |
| 4 | Virtual interactions with manufacturer sales reps (i.e., email from sales rep, video call, etc.) | **○** | **○** |
| 5 | Manufacturer websites | **○** | **○** |
| 6 | Presentation in convention, seminar, symposium, etc. | **○** | **○** |
| 7 | VIN (Veterinary Information Network) [SHOW IN US ONLY] | ○ | ○ |
| 8 | Social media | **○** | **○** |
| 9 | Printed brochures, documentation, or other information from manufacturer | **○** | **○** |
| 10 | Digital brochures, documentation, or other information from manufacturer | ○ | ○ |
| 11 | Distributor reps | **○** | **○** |
| 12 | Online webinars provided by manufacturers | **○** | **○** |
| 13 | Key opinion leaders / thought leaders / specialists | **○** | **○** |
| 14 | [SHOW IN UK ONLY:] NOAH Compendium / [SHOW IN EX-UK COUNTRIES:] National compendium / formulary | **○** | **○** |
| 91 | Other (please specify) | **○** | **○** |
| 99 | None [EXCLUSIVE] | **○** | **○** |

SECTION B: VACCINATION COMPLIANCE

OBJECTIVE – Understand vaccination compliance

- - - Compliance measures – QB1
    - Factors influencing compliance of owners bringing cat to clinic​ – QB2, QB3
    - Encouraging compliance of bringing cat to clinic – QB4, QB4a, QB5, QB6, QB7, QB8
    - Compliance with vaccination recommendations once in clinic – QB9
    - Vaccination experience in clinic including challenges to compliance – QB10, QB11
    - Additional tools/resources to improve vaccine discussions and/or compliance – QB12

Next, we would like to understand more about vaccine compliance among your cat owner clients.

QB1. How does your clinic currently monitor and/or track cat owner compliance with vaccinations? Please select all that apply.

PGR: RANDOMIZE LIST, BUT KEEP ‘OTHER, SPECIFY’ LAST

| 1 | Track the percentage of active cat patients that are up-to-date on vaccinations in the clinic database / practice information management system (PIMS) |
| --- | --- |
| 2 | Track the percentage of cat owners accepting vaccine recommendations during vaccine appointments |
| 3 | Proactively check the vaccination status of every cat who comes to the clinic (even for non-vaccine appointments) to advise on vaccination when needed |
| 91 | Other, please specify [ANCHOR] |
| 99 | We do not typically measure / track any parameters of cat vaccination compliance [ANCHOR] |

QB2. What do you think are the **most motivating** factors for your cat owner clients to **bring** their cats into the clinic to be vaccinated? Select up to 5 factors.

PGR: RANDOMIZE LIST, BUT KEEP ‘OTHER, SPECIFY’ AND ‘DON’T KNOW’ LAST

| 1 | Want to keep cat healthy / protect against disease |
| --- | --- |
| 2 | Their cat’s lifestyle (i.e., goes outdoors, travels, boards) |
| 3 | What they have read online / the internet |
| 4 | What they have seen on social media |
| 5 | What they have read in magazines, books or other print |
| 6 | What they have seen on television |
| 7 | Local vaccination requirement / law |
| 8 | Friend / family influence |
| 9 | Past experience with a sick cat |
| 10 | Veterinarian’s recommendation to get vaccinated |
| 11 | Prompted by the vet clinic that cat is due for a vaccine (e.g., via reminder postcard, phone call, etc.) |
| 12 | Recommended at time of adoption by shelter / breeder / pet shop |
| 91 | Other, please specify:____________________ |
| 99 | Don’t know [EXCLUSIVE] |

QB3. What do you believe are the **most common** reasons why cat owners may **not** regularly take their cats to the clinic for vaccinations? Select up to 5 responses.

PGR: RANDOMIZE LIST, BUT KEEP ‘OTHER, SPECIFY’ AND ‘DON’T KNOW’ LAST

| **1** | Cannot get cat into a cat carrier |
| --- | --- |
| **2** | Cat gets too stressed with transportation to the clinic |
| **3** | Think it is too expensive |
| **4** | Think if cat is healthy there is no need for vaccines |
| **5** | Think vaccines are not needed because cat is an indoor only cat |
| **6** | Think vaccines are not needed because cat is not around other animals |
| **7** | Concern about vaccine safety / side effects |
| **8** | Don’t have enough information about why their cat needs vaccines |
| **9** | Vaccines were not recommended for their cat by a veterinarian |
| **10** | Don’t believe in / opposed to vaccines for their cat |
| **11** | Think vaccinations are painful for the cat |
| **12** | Believe the experience at the clinic would be too stressful on their cat |
| **13** | Too busy / does not have time to take cat for vaccinations |
| **14** | Forget cat is due for vaccines |
| **15** | Prefer alternative medicine for their cats |
| **91** | Other, please specify |
| **99** | Don’t know **[**EXCLUSIVE] |

QB~~4~~. What do you feel would be helpful for your clients in getting them to bring their cat to the clinic for vaccinations? *Please be as specific and detailed as possible when answering.*

TEXT BOX

QB5. Which of the following do you/does your clinic do to encourage cat owner clients to bring their cat to the clinic for vaccinations? Select all that apply.

PGR: RANDOMIZE LIST, BUT KEEP ‘OTHER SPECIFY’ AND ‘NONE OF THE ABOVE’ LAST

| 1 | Vaccination reminders (via postcard, phone call, text, email, etc.) |
| --- | --- |
| 2 | Share educational materials on importance of vaccination for cats |
| 3 | Offer discounts or promotions on vaccinations |
| 4 | Maintain a website where cat owners can find information about cat vaccinations |
| 5 | Online booking of vaccine appointments |
| 6 | Offer mobile clinic service / in-home appointments |
| 7 | Offer wellness and/or health plan |
| 8 | Community outreach efforts to educate about cat vaccination (such as attending community events, etc.) |
| 9 | Forward book vaccine appointment during check-ups or other visits |
| 10 | Offer a cat friendly clinic experience (i.e., cat-only waiting rooms, cat-friendly handling, etc.) |
| 11 | Educate owners on carrier use and transporting the cat |
| 12 | Check vaccination records of all pets in the households during any pet’s visit and inform the owner if a cat is due for a vaccine |
| 13 | Offer therapies to help reduce appointment stress (i.e., pheromones, sedatives, etc.) |
| 14 | Advertise to raise awareness about vaccinations (i.e., ad in the community newspaper, billboard or sign in front of clinic, etc.) |
| 15 | Posts about feline vaccination on clinic’s social media channels |
| 16 | Recorded telephone messages that play while clients are on hold that remind them to bring in their pets for vaccination |
| 91 | Other, please specify:___________ [ANCHOR] |
| 99 | Nothing [ANCHOR; EXCLUSIVE] |

ASK QB5a IF QB5=2

QB5a. Base=share educational materials on importance of cat vaccinations

Which of the following types of vaccination educational materials do you use **most often** for cat owner clients? Select up to 3 responses.

PGR: RANDOMIZE LIST, BUT KEEP ‘OTHER, SPECIFY’ LAST

| 1 | Printed materials offered in the clinic (brochures, pamphlets, handouts, materials from industry partners, etc.) |
| --- | --- |
| 2 | Social media posts |
| 3 | Emails |
| 4 | Digital materials on clinic website page |
| 5 | Printed materials mailed to clients (mailers, postcards, etc.) |
| 6 | Text messages |
| 9 | Other, please specify:___________ [ANCHOR] |

PGR: SKIP QB6 IF QB5=99 (NOTHING)

QB6. Base=currently do to encourage cat owners to bring cat to clinic

How effective are each of the following in encouraging your cat owner clients to bring their cat to the clinic for vaccinations?

|  |  | **Not effective at all**  **1** | **2** | **3** | **4** | **5** | **6** | **Extremely effective**  **7** | **Not sure**  9 |
| --- | --- | --- | --- | --- | --- | --- | --- | --- | --- |
|  | [SHOW CHOICES SELECTED IN QB5] |  |  |  |  |  |  |  |  |

QB7. Looking ahead to the next 12 months, do you / does your clinic plan to [IF QB5=NOTHING, SHOW: start any / IF QB5≠ NOTHING, SHOW: expand or increase] efforts to encourage cat owner clients to bring their cat to the clinic for vaccinations?

| 1 | Yes |
| --- | --- |
| 2 | No |
| 3 | Maybe |

PGR: ONLY ASK QB8 IF QB7=NO

QB8. Base=not going to start/expand efforts to get owners to bring cat to clinic for vaccinations

Why do you / does your clinic not plan to [IF QB5=NOTHING, SHOW: start any / IF QB5≠ NOTHING, SHOW: expand or increase] efforts to encourage your cat owner clients to bring their cat to the clinic for vaccinations? Select all that apply.

PGR: RANDOMIZE LIST, BUT KEEP ‘OTHER SPECIFY’ LAST

| 1 | No need / the current approach is working fine |
| --- | --- |
| 2 | No time to implement new processes |
| 3 | Unsure what steps to take or how to improve |
| 4 | Lack of buy-in or support from clinic staff |
| 5 | Lack of buy-in or support from clinic management / ownership |
| 6 | Cat vaccinations are not a focus or priority for the clinic |
| 7 | Cat vaccinations do not generate enough revenue for the clinic to invest in such efforts |
| 8 | Have tried to improve compliance in the past and it did not work |
| 91 | Other, please specify:_________ [ANCHOR] |

Now for the next set of questions we would like you to think about your cat owner clients who **do bring** their cats into the clinic for wellness visits.

B9. Thinking about the cats you personally see that do come to the clinic, what percentage of cat owner clients comply with your vaccination recommendations? Please answer for both core and non-core vaccinations.

- By CORE, we mean those vaccines that all cats should receive regardless of their geography or lifestyle.
- NON-CORE are those vaccines that are offered based on the risks associated with the lifestyle of the cat or where they live.

|  |  | % of cat owners who comply with your vaccine recommendations |
| --- | --- | --- |
| 1 | Core vaccines |  |
| 2 | Non-Core vaccines |  |

QB10. When a cat owner is at the clinic for an appointment with their cat, what is typically discussed with the owner about vaccination? Select all that apply.

[RANDOMIZE LIST, BUT KEEP ‘OTHER’ AND ‘NONE’ LAST]

| 1 | The need for and/or benefits of vaccination |
| --- | --- |
| 2 | What the vaccines protect against (i.e., talk about the disease) |
| 3 | Safety of vaccines |
| 4 | Efficacy of vaccines |
| 5 | What vaccines their cat is receiving (i.e., what the cat is due for) |
| 6 | How the vaccine will be administered |
| 7 | The brand and/or manufacturer of the vaccine |
| 8 | Cost of the vaccine |
| 9 | How long the vaccine will last / when the next booster is needed |
| 10 | Unique features of the vaccine (e.g., low volume dose, non-adjuvanted, etc.) |
| 11 | Proactively ask owners if they have any questions related to vaccination |
| 12 | After visit instructions (set expectations for any behavioral changes, monitoring for side effects, etc.) |
| 91 | Other, please specify |
| 99 | Nothing; vaccination is not typically discussed with cat owner clients [EXCLUSIVE] |

QB11. What challenges or difficulties, if any, do you face in discussing and/or recommending vaccines to your cat owner clients during appointments? Select all that apply.

[RANDOMIZE LIST, BUT KEEP ‘OTHER’ AND ‘NONE’ LAST]

| 1 | Addressing misinformation the cat owner has heard (from internet, breeder, etc.) |
| --- | --- |
| 2 | Discussing cost / navigating cost concerns expressed by cat owner |
| 3 | Clients’ lack of trust in vet recommendations |
| 4 | Clients’ poor understanding of the importance of vaccination |
| 5 | Lack of client compliance with vaccine recommendations |
| 6 | Clients who are opposed to vaccines in general / anti-vaccination |
| 7 | Not enough time during patient visit to discuss vaccination |
| 8 | Not enough consistency among all clinic team members in how and/or what vaccine benefits are communicated to cat owners |
| 9 | Client’s lack of interest in learning about vaccinations for their cats |
| 91 | Other, please specify:_______________ [ANCHOR] |
| 99 | Nothing / no challenges or difficulties [ANCHOR; EXCLUSIVE] |

QB12. What tools, trainings, or resources would be helpful for you, your clinic and/or your cat owner clients when discussing and/or recommending vaccines during appointments? *Please be as specific and detailed as possible when answering.*

TEXT BOX

SECTION C: VACCINE BRAND USAGE & DRIVERS FOR USE

OBJECTIVE – Understand current approach towards vaccinating cats

- - - Brands used – QC1

OBJECTIVE – Understand drivers for brand selection and brand switching

- - - Drivers for feline vaccine brand selection – QC2a, QC2b
    - Drivers for brand switching​ – QC3, QC3a, QC3b, QC4, QC4a, QC4b, QC5

The next set of questions deal with your experiences with different vaccine brands for CATS.

QC1. Which of the following BEST describes your experience with each of the following vaccine brands for ***CATs***? Please select one response for each vaccine brand shown*.*

|  | Currently use this vaccine brand in **cats** | Have used in the past for **cats**, but do NOT currently use **for cats** | Familiar with brand but have never used **for cats** | Not familiar with this brand |
| --- | --- | --- | --- | --- |
| INSERT RELEVANT BRANDS FOR COUNTRY |  |  |  |  |

QC2a. How **important** are each of the following when deciding which vaccine brand(s) to use for your **CAT** patients?

| **[RANDOMIZE LIST]** | Not important at all  1 | 2 | 3 | 4 | 5 | 6 | Extremely important  7 |
| --- | --- | --- | --- | --- | --- | --- | --- |
| High degree of efficacy | **1** | **2** | **3** | **4** | **5** | **6** | **7** |
| Few side effects / adverse reactions | **1** | **2** | **3** | **4** | **5** | **6** | **7** |
| Covers the latest strains of disease | **1** | **2** | **3** | **4** | **5** | **6** | **7** |
| Offers core / non-core combination products (e.g., HCP + FeLV) to minimize number of injections given | **1** | **2** | **3** | **4** | **5** | **6** | **7** |
| Offers a complete range of vaccines in the brand portfolio (products to cover all relevant diseases) | **1** | **2** | **3** | **4** | **5** | **6** | **7** |
| Offer non-adjuvanted options | **1** | **2** | **3** | **4** | **5** | **6** | **7** |
| Offer low volume options (i.e. 0.5ml) | **1** | **2** | **3** | **4** | **5** | **6** | **7** |
| Low risk of reversion to virulence | **1** | **2** | **3** | **4** | **5** | **6** | **7** |
| Offer long duration of immunity options (i.e., up to 3 years) | **1** | **2** | **3** | **4** | **5** | **6** | **7** |
| Endorsed by key opinion leader / thought leader / specialist | **1** | **2** | **3** | **4** | **5** | **6** | **7** |
| Recommended by other vets / colleagues | **1** | **2** | **3** | **4** | **5** | **6** | **7** |
| Supported by scientific advice / guidelines / evidence | **1** | **2** | **3** | **4** | **5** | **6** | **7** |
| Offers the newest vaccine technology / innovation | **1** | **2** | **3** | **4** | **5** | **6** | **7** |

QC2b. And, how **important** are each of the following manufacturer offerings when deciding which vaccine brand(s) to use in your practice?

| **[RANDOMIZE LIST]** | Not important at all  1 | 2 | 3 | 4 | 5 | 6 | Extremely important  7 |
| --- | --- | --- | --- | --- | --- | --- | --- |
| Price | **1** | **2** | **3** | **4** | **5** | **6** | **7** |
| Promotions, discounts, or rebates | **1** | **2** | **3** | **4** | **5** | **6** | **7** |
| Reliable availability / no or limited supply issues | **1** | **2** | **3** | **4** | **5** | **6** | **7** |
| Offers a vaccine guarantee | **1** | **2** | **3** | **4** | **5** | **6** | **7** |
| Offers a commercial loyalty program | **1** | **2** | **3** | **4** | **5** | **6** | **7** |
| Medical/product technical support | **1** | **2** | **3** | **4** | **5** | **6** | **7** |
| Offers general educational resources / training for clinic staff | **1** | **2** | **3** | **4** | **5** | **6** | **7** |
| Printed/physical client educational materials | **1** | **2** | **3** | **4** | **5** | **6** | **7** |
| Digital client educational materials | **1** | **2** | **3** | **4** | **5** | **6** | **7** |
| Other value-added offerings (e.g., free syringes, etc.) | **1** | **2** | **3** | **4** | **5** | **6** | **7** |
| Relationship with sales representative | **1** | **2** | **3** | **4** | **5** | **6** | **7** |
| Ease of working with manufacturer/company | **1** | **2** | **3** | **4** | **5** | **6** | **7** |
| Offers other feline preventative healthcare products (i.e., parasiticides) along with vaccines | **1** | **2** | **3** | **4** | **5** | **6** | **7** |
| Offers feline-focused educational training programs to the clinic (i.e., feline vaccines, feline-friendly handling) | **1** | **2** | **3** | **4** | **5** | **6** | **7** |

QC3. When was the last time, if ever, that you switched vaccine brands that you recommend or use for your **CAT** patients?

| 1 | Within the past 12 months |
| --- | --- |
| 2 | Between 12 and 24 months ago |
| 3 | More than 24 months ago |
| 4 | Never |

PGR: ONLY ASK QC3a-QC3b IF QC3=1 OR 2

QC3a. Base=switched vaccine brand in past 24 months

Why did you switch vaccine brands for your **CAT** patients?  Select all that apply.

[RANDOMIZE; ANCHOR “OTHER, PLEASE SPECIFY” LAST]

| 1 | Price |
| --- | --- |
| 2 | Promotional pricing program / incentives / rebates |
| 3 | Relationship with sales rep |
| 4 | Bundling offers with other products from the same manufacturer |
| 5 | Better safety profile |
| 6 | Supply shortages with previous manufacturer |
| 7 | New innovation offered |
| 8 | Manufacturer offers vaccine guarantee |
| 9 | Manufacturer support items (free syringes, pet health records, client education materials, etc.) |
| 10 | Can purchase through distributor of my choice |
| 11 | Offers core / non-core combination products (e.g., HCP + FeLV) to minimize number of injections given |
| 12 | Offers a complete range of vaccines in the brand portfolio (products to cover all relevant diseases) |
| 13 | Better efficacy profile |
| 14 | Offers non-adjuvanted options |
| 15 | Longer duration of immunity (i.e., 3 years) |
| 16 | Brand was recalled/withdrawn |
| 17 | Decision was made by clinic management |
| 18 | Advised by key opinion leader / thought leader / specialist |
| 19 | Recommended by other vets / colleagues |
| 20 | New scientific advice / guidelines / evidence |
| 21 | Offers other feline preventative healthcare products (i.e., parasiticides) along with vaccines |
| 22 | Offers feline-focused educational training programs to the clinic (i.e., feline vaccines, feline-friendly handling) |
| 23 | Offers general educational resources/training for clinic staff (i.e. not product specific) |
| 91 | Other, please specify:________________ [ANCHOR] |

QC3b. Base=switched vaccine brand in past 24 months

Which vaccine brands have you started using and which brands have you stopped using in the past [IF QC3=1 SHOW: 12 months / IF QC3=2 SHOW: 24 months] for your **CAT** patients?

| **Brands Stopped Using** |
| --- |
| [INSERT BRANDS CURRENTLY USING OR EVER USED IN QC1) |

| **Brands Started Using** |
| --- |
| [INSERT BRANDS CURRENTLY USING OR EVER USED IN QC1) |

QC4. Looking ahead to the next 12 months, do you plan to switch vaccine brands that you recommend or use for your **CAT** patients?

| 1 | Yes |
| --- | --- |
| 2 | No |
| 3 | Maybe |

PGR: ONLY ASK QC4a-QC4b IF QC4=1

QC4a. Base=planning to switch vaccine brand in next 12 months

Why are you planning to switch vaccine brands for your **CAT** patients in the next year?  Select all that apply.

[RANDOMIZE; ANCHOR “OTHER, PLEASE SPECIFY” LAST]

| 1 | Price |
| --- | --- |
| 2 | Promotional pricing program / incentives / rebates |
| 3 | Relationship with sales rep |
| 4 | Bundling offers with other products from the same manufacturer |
| 5 | Better safety profile |
| 6 | Supply shortages with current manufacturer |
| 7 | New innovation offered |
| 8 | Manufacturer offers vaccine guarantee |
| 9 | Manufacturer support items (free syringes, pet health records, client education materials, etc.) |
| 10 | Can purchase through distributor of my choice |
| 11 | Offers core / non-core combination products (e.g., HCP + FeLV) to minimize number of injections given |
| 12 | Offers a complete range of vaccines in the brand portfolio (products to cover all relevant diseases) |
| 13 | Better efficacy profile |
| 14 | Offers non-adjuvanted options |
| 15 | Offers longer duration of immunity options (i.e., 3 years) |
| 16 | Brand was recalled/withdrawn |
| 17 | Decision was made by clinic management |
| 18 | Advised by key opinion leader / thought leader / specialist |
| 19 | Recommended by other vets / colleagues |
| 20 | New scientific advice / guidelines / evidence |
| 21 | Offers other feline preventative healthcare products (i.e., parasiticides) along with vaccines |
| 22 | Offers feline-focused educational training programs to the clinic (i.e., feline vaccines, feline-friendly handling) |
| 23 | Offers general educational resources/training for clinic staff (i.e. not product specific) |
| 91 | Other, please specify:________________ [ANCHOR] |

QC4b. Base=planning to switch vaccine brand in next 12 months

Which vaccine brands are you are planning to start using and which brands are you planning to stop using in the next 12 months for your **CAT** patients?

| **Brands Planning to Stop Using** |
| --- |
| [INSERT BRANDS CURRENTLY USING IN QC1) |

| **Brands Planning to Start Using** |
| --- |
| [INSERT BRANDS FAMILIAR WITH IN QC1) |
| Not sure |

PGR: ONLY ASK QC5 IF QC3=3 OR 4 AND QC4=2 OR 3 OR 4

QC5. Base=did not switch vaccine brand in past 24 months nor plan to in next 12 months

Which of the following factors would be **most motivating** for you to consider switching the brand of vaccines you use for your **CAT** patients? Select up to 5 responses.

[RANDOMIZE]

| 1 | Price |
| --- | --- |
| 2 | Promotional pricing program / incentives / rebates |
| 3 | Relationship with sales rep |
| 4 | Bundling offers with other products from the same manufacturer |
| 5 | Better safety profile |
| 6 | Low risk of supply shortages |
| 7 | New innovation offered |
| 8 | Manufacturer offers vaccine guarantee |
| 9 | Manufacturer support items (free syringes, pet health records, client education materials, etc.) |
| 10 | Can purchase through distributor of my choice |
| 11 | Offers core / non-core combination products (e.g., HCP + FeLV) to minimize number of injections given |
| 12 | Offers a complete range of vaccines in the brand portfolio (products to cover all relevant diseases) |
| 13 | Better efficacy profile |
| 14 | Offers non-adjuvanted options |
| 15 | Offers longer duration of immunity options (i.e., 3 years) |
| 16 | No history of recalls/withdrawals |
| 17 | Decision made by clinic management |
| 18 | Advised by key opinion leader / thought leader / specialist |
| 19 | Recommended by other vets / colleagues |
| 20 | New scientific advice / guidelines / evidence |
| 21 | Offers other feline preventative healthcare products (i.e., parasiticides) along with vaccines |
| 22 | Offers feline-focused educational training programs to the clinic (i.e., feline vaccines, feline-friendly handling) |
| 23 | Offers general educational resources/training for clinic staff (i.e. not product specific) |
| 91 | Other, please specify:________________ [ANCHOR] |
| 99 | Nothing; I would never consider switching vaccine brands for my CAT patients [ANCHOR; EXCLUSIVE] |

Finally, to wrap up we have just one more question.

QE1. So far, this survey has been focused on cats. However, for this question please now also think about your dog patients.

When it comes to deciding which vaccines to use, do you prefer to use the same vaccine manufacturer for **both** your cat and dog patients?

[RANDOMIZE]

| 1 | Yes |
| --- | --- |
| 2 | No |
| 3 | No preference |

CLOSE: Thank you! Those are all the questions we have for you today.
