# Supplementary material for: Drivers, Barriers and Unmet Needs Affecting Feline Vaccination Compliance: Insights from a Global Survey of Cat Owners and Veterinarians
Source: Vet Sci. 2026 Jun 30;13(7):646. doi: 10.3390/vetsci13070646 (PMC13431493; doi:10.3390/vetsci13070646)
Supplement: Supplementary file 1 [file vetsci-13-00646-s001.zip › Global Feline Vaccines_CAT OWNERS_Survey.docx]

**Supplemental Questionnaire 1- Cat Owner Survey**

**FOR INTERNAL USE ONLY – NOT SHOWN TO RESPONDENTS**

- **TARGET RESPONDENTS / RESPONDENT QUALIFICATIONS**

**A random representative sampling of consumers that meet the following criteria:**

- Own one or more cats
- Greater than 18 years of age
- Primary decision-maker or share in the decision-making process regarding the cat’s health care
- Not affiliated with any animal health, market research, pharmaceutical, or veterinary companies
- Representative mix of cat owner demographics, geography, gender, age, household income, etc.
- **METHODOLOGY:** Online survey
  - - Budgeted Length: 10 minutes **CURRENT ESTIMATE=16 MINUTES**

**SURVEY BEGINS HERE FOR RESPONDENTS. ANY TEXT FOUND INSIDE BRACKETS [] OR IN BOLD BLUE FONT ARE FOR INTERNAL USE ONLY AND WILL NOT BE SEEN BY RESPONDENTS.**

**WELCOME SCREEN**

Thank you for your interest in our study. Your opinions are very important to us!

This is a country-wide study designed to represent consumers from varying situations. For this reason, the first section of this survey consists of a few classification questions.

If you fit into one of the groups of consumers that are needed for this research, you will be given the option to participate in this survey. If you do not fit into one of the groups of consumers currently needed to complete the survey, we want you to know that we appreciate your interest in the study and hope that you will be able to participate in future research.

It will only take a few minutes to determine if you fit into one of the groups needed for this study.

In accordance with standard market research practices your identity will remain confidential! Your responses will not be linked with your name and your information will be used for research purposes only.

When you are ready to begin, please click "**Continue**".

**INTEGRITY QUESTION**

Honest answers are important to the integrity of the research process. As such, please take the time necessary to provide thorough and thoughtful answers to this survey. If you attempt to complete the survey in an unreasonable amount of time or answer in nonsensical ways, you may be disqualified.

Before you begin, please check the box indicating agreement with our survey integrity policy.

- I agree to carefully read and respond as accurately as possible to all questions within this survey.

**[PROGRAMMER: RESPONDENT MUST CHECK THE BOX BEFORE BEING ALLOWED TO MOVE ON TO THE SURVEY]**

**SCREENING QUESTIONS**

**OBJECTIVE – Screening criteria, demos – Section S – S1, S2, S3, S4, S5, S6, S7**

QS1. In which state/province/region do you currently live?

**[SHOW DROP DOWN LIST OF APPROPRIATE STATES/REGIONS BASED ON COUNTRY.]**

QS2. Which of the following includes your current age?

| <18 years old | 1 | **[TERMINATE]** |
| --- | --- | --- |
| 18-24 years old | 2 |  |
| 25-34 years old | 3 |  |
| 35-44 years old | 4 |  |
| 45-54 years old | 5 |  |
| 55-64 years old | 6 |  |
| 65+ years old | 7 |  |

QS4. Do you work in or are you employed by any of the following types of businesses or professions?

**[MULTI-PUNCH]**

| Veterinarian /Veterinary clinic or hospital | 1 | **[TERMINATE]** |
| --- | --- | --- |
| The manufacturing, distribution or research of animal health care products | 2 | **[TERMINATE]** |
| Advertising, marketing or marketing research agency | 3 | **[TERMINATE]** |
| None of the above | 96 | **[CONTINUE]** |

QS5. Which, if any, of the following pets do you currently have? Please select all that apply.

**[MULTI-PUNCH]**

| Dog | 1 |
| --- | --- |
| Cat | 2 |
| Rabbit | 3 |
| Some other type of pet | 98 |
| Do not have a pet | 99 |

**[IF S5 = 2 CONTINUE, OTHERWISE TERMINATE] [CHECK QUOTAS - MINIMUM OF 100 CAT-ONLY HOUSEHOLDERS AND 100 CAT+DOG HOUSEHOLDS PER COUNTRY (MINIMUM OF 75 EACH IN BELGIUM, NO QUOTAS IN DENMARK)]**

QS6. How many **CATs** do you currently own?

| Number of cats | ____ |
| --- | --- |

**[ACCEPTED RANGE = 0 – 99] [TERMINATE IF S6=0 OR IF S6>5]**

QS7. What role do you play in decisions related to the health care products /services purchased for your **CAT(s)**? Would you say that you…?

**[SINGLE PUNCH]**

| Are the sole decision maker | 1 | **[Continue]** |
| --- | --- | --- |
| Share in the decision-making responsibility | 2 | **[Continue]** |
| Have little to no role in decisions regarding health care products/services purchased for your cat(s) | 3 | **[TERMINATE]** |

**Invitation: [DO NOT SHOW HEADING]**

**[IF RESPONDENT QUALIFIES, SHOW:]**

Thank you for taking a few minutes to answer our questions! You are eligible to participate in our study.

**Termination text: [DO NOT SHOW HEADING]**

**[IF RESPONDENT SCREENS OUT, SHOW:]**

Unfortunately, you do not fit into one of the groups of people that we are currently seeking to take part in this study. However, your opinions are important to us, so we hope that you will be able to participate in a future study. Thank you again and have a good day!

**reporting of adverse events**

**PLACEHOLDER FOR UPDATED AE STATEMENT FROM MSD**

**QUESTIONNAIRE SUMMARY**

QA1-A4 – cat demographics, cat health/wellbeing questions –

ALL RESPONDENTS

QA5– Cat vaccinated yes or no – ALL RESPONDENTS

QC1-C2– cat not vaccinated reasons why, what vet clinic could offer to overcome challenges;

QD2-QD2b drivers to start getting cat vaccinated–

CAT NOT VACCINATED

QB1-B6– cat vaccinated -

when, why, influences, what vet clinic could offer to overcome challenges; QD2-QD2b drivers to continue getting cat vaccinated –

CAT VACCINATED

QD3- D7 cat vaccination awareness; familiarity; sources of information–

ALL RESPONDENTS

Section F- segmentation

Section E – cat owner demos – ALL RESPONDENTS

**Questionnaire**

**SECTION A: CAT DEMOGRAPHICS, HEALTH AND WELL-BEING**

**OBJECTIVE – Cat owners’ understanding of cat’s health and well-being**

- **QA1, QA40-QA80 – cat demographics (for profiling)**
- **QA2/QA3 – what is important for keeping cats healthy**
- **QA4/QA5 – vet visits - how often, what they go to the vet for**

Today’s survey will be about your **cat(s)**.

**QA1.** What **[IF QS6=1, SHOW:** is **/ IF QS6>1, SHOW:** are**]** the **[IF QS6=1, SHOW:** name **/ IF QS6>1, SHOW:** names**]** of your **[IF QS6=1, SHOW:** cat **/ IF QS6>1, SHOW:** cats**]**?

**[MANDATORY TEXT BOX(ES). SHOW SAME NUMBER OF BOXES AS CATS IN QS6]**

**QA40.** Where did you get your **[IF QS6=1, SHOW:** cat **/ IF QS6>1, SHOW:** cats**]**? **[IF QS6>1, SHOW:** *Please answer for each of your cats.***]**

| **[RANDOMIZE LIST]** | **[INSERT CAT NAMES FROM QA1, 1 PER COLUMN]** |
| --- | --- |
| Animal shelter / pound |  |
| Breeder |  |
| Breed rescue organization |  |
| Classified Ad |  |
| Friend / family / neighbor |  |
| Found / stray |  |
| From own litter |  |
| Pet store |  |
| Veterinarian |  |
| Other, please specify **[ANCHOR LAST]** |  |

**QA50.** What is the age range of your **[IF QS6=1, SHOW:** cat **/ IF QS6>1, SHOW:** cats**]**?

|  | **[INSERT CAT NAMES FROM QA1, 1 PER COLUMN]** |
| --- | --- |
| Less than 1 year old |  |
| 1-6 years of age |  |
| 7-10 years of age |  |
| 11~~+~~ years of age |  |

**QA60.** What **[IF QS6=1, SHOW:** is your cat’s breed **/ IF QS6>1, SHOW:** are your cats’ breeds**]**?

|  | **[INSERT CAT NAMES FROM QA1, 1 PER COLUMN]** |
| --- | --- |
| Pure breed |  |
| Mixed breed |  |
| Not sure / don’t know |  |

**QA70.** Please select which of the following best describes your **[IF QS6=1, SHOW:** cat’s lifestyle **/ IF QS6>1, SHOW:** cats’ lifestyles**]** …

|  | **[INSERT CAT NAMES FROM QA1, 1 PER COLUMN]** |
| --- | --- |
| Lives indoors and never goes outside |  |
| Lives indoors and rarely goes outside |  |
| Lives indoors and routinely goes outside |  |
| Lives exclusively outdoors |  |

**QA80.** Please indicate if your **[IF QS6=1, SHOW:** cat ever does **/ IF QS6>1, SHOW:** cats ever do**]** any of the following…

| **[RANDOMIZE LIST]** | **[INSERT CAT NAMES FROM QA1, 1 PER COLUMN]** |
| --- | --- |
| Travels with you |  |
| Is boarded (in a kennel/cattery) |  |
| Participates in cat shows / competitions |  |
| None of these **[ANCHOR LAST; EXCLUSIVE]** |  |

**QA2.*(QA1vet)** In your opinion, what are the top 3 most important things you can do to keep your **[IF QS6=1, SHOW:** cat **/ IF QS6>1, SHOW:** cats] healthy? *Please use a separate line for each response.*

**[PRG: SHOW 3 TEXT BOXES]**

**QA3.** For each group of **[X]** options, please indicate which is the most important to you and which is the least important to you, to keep your **[IF QS6=1, SHOW:** cat **/ IF QS6>1, SHOW:** cats] healthy and well.

| **Least Important** |  | **Most Important** |
| --- | --- | --- |
|  | Option 1 |  |
|  | Option 2 |  |
|  | Option 3 |  |
|  | Option 4 |  |

| **1** | Annual veterinary visit |
| --- | --- |
| **2** | Balanced and nutritious diet |
| **3** | Toys to play with |
| **4** | Flea/tick prevention |
| **5** | Internal parasite prevention (heartworm, tapeworm, roundworm, etc.) |
| **6** | Initial kitten vaccines |
| **7** | Vaccines/boosters for adult cat |
| **8** | Regular grooming |
| **9** | Dental care / cleaning teeth |
| **10** | Time outdoors |
| **11** | Maintaining a healthy weight |
| **12** | Monitoring for signs of illness / injury |
| **13** | Social interaction with people |
| **14** | Social interaction with other animals |
| **15** | A safe comfortable environment |
| **16** | Spaying / neutering |

**QA4.** When was the last time you took your **[IF QS6=1, SHOW:** cat **/ IF QS6>1, SHOW:** cats**]** to the vet? **[IF QS6>1, SHOW:** *Please answer for each of your cats.***]**

|  | **[INSERT 1 COLUMN FOR EACH CAT NAME FROM QA1]** |
| --- | --- |
| Within the last 12 months | **1** |
| In the last 1-2 years | **2** |
| More than 2 years ago | **3** |
| Never visited the veterinarian | **4** |

**ASK QA5 IF QA4≠4**

**QA5**. Base = cat has been to veterinarian

When you think of the reasons you have ever taken your **[IF QS6=1, SHOW:** cat **/ IF QS6>1, SHOW:** cats**]** to the vet, which of the following reasons apply? **[IF QS6>1, SHOW:** *Please answer separately for each of your cats.]*

*Select all that apply.*

**[RANDOMIZE LIST]**

|  |  | **[INSERT 1 COLUMN FOR EACH CAT NAME FROM QA1]** |
| --- | --- | --- |
| **1** | Routine check-up / wellness exam (exam only; **excluding vaccinations**) |  |
| **2** | Routine check-up / wellness exam **including vaccinations** |  |
| **3** | Vaccination(s) only appointment (excluding check-up / examination) |  |
| **4** | Treatment for an illness or injury |  |
| **5** | Parasite prevention / treatment (such as flea/tick, tapeworm, roundworm etc.) |  |
| **6** | Behavioral concerns |  |
| **7** | Nutritional/dietary reasons |  |
| **8** | Senior care for age-related issues |  |
| **9** | Spaying/neutering |  |
| **10** | Dental care |  |
| **11** | Management of a chronic disease |  |
| **91** | Other, please specify **[ANCHOR]** |  |

**[PRG: CREATE HIDDEN VARIABLE; FLAG CAT AS VACCINATED IF QA5=2 OR 3 AND FLAG CAT AS NON-VACCINATED IF QA4=4 OR QA5≠2 OR 3**

**PGR: CREATE HIDDEN SURVEYPET VARIABLE WITH CAT NAME TO USE FOR REMAINDER OF THE QUESTIONNAIRE. IF QS6>1, RANDOMLY SELECT ONE CAT FROM THE HH TO ANSWER THE REMAINING QUESTIONS USING THE FOLLOWING LOGIC:**

- **IF MAJORITY OF CATS CLASSIFIED AS EITHER VACCINATED OR NON-VACCINATED, RANDOMLY ASSIGN ONE CAT NAME FROM THE GROUP WITH THE MAJORITY OF CATS**
- **IF CATS ARE EVENLY SPLIT BETWEEN VACCINATED AND NON-VACCINATED, USE LEAST FILL TO ASSIGN CAT NAME USING THE FOLLOWING QUOTA TARGETS: 50% VACCINATED, 50% NON-VACCINATED]**

**IF QS6>1, SHOW:** For the remainder of the survey, please think of **[SURVEYPET]** when considering your responses.

**SECTION B: VACCINATED CATS [A5_ SURVEYPET=2 OR 3]**

**OBJECTIVE – Vaccination usage / influences / information**

- **QB1, QB2 – age(s) cat received vaccine and how long ago**
- **QB3 – what influenced decision to vaccinate.**
- **QB4 – information cat owner wants to know about vaccines**
- **QB5 – challenges getting cat to clinic**

QB1. Which of the following describes **[SURVEYPET]**? Select all that apply.

| **1** | Received at least one vaccine as a kitten *(when cat was 1 year old or less)* |
| --- | --- |
| **2** | Received at least one vaccine as an adult cat *(when cat was >1 year old)* |

QB2. When was the last time **[SURVEYPET]** received a vaccine?

| **1** | Within the last 12 months |
| --- | --- |
| **2** | In the last 1-2 years |
| **3** | In the last 2-3 years |
| **4** | More than 3 years ago |
| **5** | I don’t know / don’t remember |

QB3 *(QB2vet). Which of the following factors influenced your decision to get **[SURVEYPET]** vaccinated? *Select all that apply.*

**[RANDOMIZE LIST]**

| **1** | Want to keep my cat healthy / protected against diseases |
| --- | --- |
| **2** | My vet’s recommendation |
| **3** | Friend/family influence |
| **4** | My cat goes outdoors |
| **5** | What I read online / the internet |
| **6** | What I read in magazines, books, other print media |
| **7** | What I read / saw on social media |
| **8** | What I saw on television |
| **9** | Vaccination is a requirement / law |
| **10** | Past experience with a sick cat |
| **11** | My cat travels and/or stays at catteries |
| **12** | Received a reminder from my vet clinic that my cat is due for a vaccine |
| **13** | Breeder’s recommendation |
| **91** | Other, please specify **[ANCHOR LAST]** |

QB4.*(QB10vet) When you are at the clinic for a vaccine appointment for **[SURVEYPET]** which of the following are you most interested in knowing when your vet is discussing vaccinations?

*Select all that apply*.

[RANDOMIZE LIST, BUT KEEP ‘OTHER’ AND ‘NONE’ LAST]

| 1 | The need for and/or benefits of vaccination |
| --- | --- |
| 2 | The diseases the vaccines protect against |
| 3 | Safety of vaccines |
| 4 | How well the vaccines protect my cat |
| 5 | What vaccines my cat is due for / receiving |
| 6 | How the vaccine will be administered to my cat |
| 7 | The brand of the vaccine and/or the company that makes the vaccine |
| 8 | Cost of the vaccine |
| 9 | How long the vaccine will last / when the next booster is needed |
| 10 | Any special features of the vaccine (e.g., dose size, ingredients, etc.) |
| 11 | Asking if I have any questions |
| 12 | After visit instructions (what to watch for with my cat’s behavior/mood, when a callback to the vet would be needed, etc.) |
| 91 | Other, please specify |
| 99 | Nothing [EXCLUSIVE] |

QB5. *(QB3vet) What are the biggest challenges to visiting your veterinarian with **[SURVEYPET]**? *Select all that apply.*

[RANDOMIZE LIST, BUT KEEP ‘OTHER’ AND ‘NONE’ LAST]

| **1** | Getting the cat into the travel carrier |
| --- | --- |
| **2** | Travelling to the veterinarian with the cat (cat is crying, yowling, vomiting, urinating, defecating, scared) |
| **3** | The cost of the veterinary service and/or products |
| **4** | The stress during the examination (cat is crying, yowling, hissing, growling, and/or trying to bite/scratch the employees) |
| **5** | Can’t find the cat when it is time to leave for the appointment (cat is hiding or is outside and can’t be found) |
| **6** | Obtaining an appointment at a convenient time for me |
| **7** | Stress of encountering other animals at the clinic |
| **91** | Other – please specify **[ANCHOR LAST]** |
| **99** | I don’t face any challenges when taking my cat to the vet **[ANCHOR LAST; EXCLUSIVE]** |

QB6*(QB4vet) What do you feel your vet clinic could offer to help overcome any challenges you face in getting your cat(s) to the clinic for vaccinations? *Please be as specific and detailed as possible when answering.*

TEXT BOX

**SECTION C: NON-VACCINATED CATS** **(A5≠2 OR 3)**

**OBJECTIVE – Understand why cats are not vaccinated**

- **QC1– reasons why not vaccinated**

**QC1.*(QB3vet)** What are the **main** reasons you have not taken **[SURVEYPET]** to the vet for vaccinations? *Select all that apply.*

**[RANDOMIZE LIST]**

| **1** | Cost of the veterinary service and/or products |
| --- | --- |
| **2** | Too difficult to get the cat into the travel carrier |
| **3** | Cat gets stressed traveling to the clinic vet (cat is hiding when it is time to leave, cat cries, yowls, vomits, is scared, etc. in the carrier) |
| **4** | Difficult to schedule an appointment that is convenient for me / no time that fits my schedule |
| **5** | Getting to the vet clinic is difficult (too far away, no transportation, etc.) |
| **6** | No reason to go / cat is healthy |
| **7** | Bad experience with other pets at the clinic |
| **8** | Afraid that the appointment / exam will be too stressful (encountering other animals,  being restrained, etc) |
| **9** | Fear vaccination will be painful for my cat |
| **10** | My cat is an indoor only cat |
| **11** | My cat is not around other animals |
| **12** | Concern about vaccine safety / side effects |
| **13** | Don’t have enough information about vaccines my cat needs |
| **14** | My vet doesn’t talk about and/or recommend vaccines |
| **15** | I don’t believe in vaccines for my cat |
| **16** | I don’t think vaccines are effective |
| **17** | I forgot cat is due for vaccine / needs vaccinations |
| **91** | Other, please specify **[ANCHOR LAST]** |

UPDATED WORDING

QC2*(QB4vet) What do you feel a vet clinic could offer to help encourage you to take your cat to the clinic for vaccinations? *Please be as specific and detailed as possible when answering.*

TEXT BOX

**SECTION D: VACCINE DRIVERS AND SOURCES OF INFORMATION**

**OBJECTIVE – Drivers for vaccination**

- **QD2, QD2a/b – drivers for getting cats vaccinated**
- **QD3/QD4/QD5 – familiarity / awareness of feline vaccines and features**
- **QDb/QD6a/QD6b/QD6c – importance of vaccine features**
- **QC7 – information sources used for vaccines**

QD1.*(QB5vet) **IF A6=3 (VACCINATED CAT):** How influential would each of the following be for you to continue getting your cat vaccinated?

**IF QA6≠3 (NON-VACCINATED CAT), SHOW:** How influential would each of the following be for you to start taking your cat(s) to the clinic for vaccines?

**[PRG REPEAT HEADINGS EVERY 5 STATEMENTS]**

|  | **[RANDOMIZE LIST]**  **[REPEAT SCALE EVERY 5 STATEMENTS]** | Not influential at all  1 | 2 | 3 | 4 | 5 | 6 | Extremely influential  7 |
| --- | --- | --- | --- | --- | --- | --- | --- | --- |
| **1** | A way to calm my cat during transportation to the clinic |  |  |  |  |  |  |  |
| **2** | A way to reduce stress during appointments |  |  |  |  |  |  |  |
| **3** | Guidance on how to transport my cat to the clinic |  |  |  |  |  |  |  |
| **4** | A way for the clinic to come to me (mobile clinic) |  |  |  |  |  |  |  |
| **5** | A community event to learn more about cat vaccinations |  |  |  |  |  |  |  |
| **6** | Lower cost / discounts / promotions |  |  |  |  |  |  |  |
| **7** | Cat-friendly features at the clinic such as a cat-only waiting room |  |  |  |  |  |  |  |
| **8** | Printed pamphlets, booklets, other materials to learn how vaccines help my cat |  |  |  |  |  |  |  |
| **9** | Information I can read online to learn how vaccines help my cat |  |  |  |  |  |  |  |
| **10** | Information on vaccine safety |  |  |  |  |  |  |  |
| **11** | A reminder from the vet clinic to schedule the vaccine appointment |  |  |  |  |  |  |  |
| **12** | The ability to book appointments myself online |  |  |  |  |  |  |  |
| **13** | A wellness plan / health plan offered by the vet clinic |  |  |  |  |  |  |  |
| **14** | Vaccine recommendations from my veterinarian |  |  |  |  |  |  |  |
| **15** | Convenient appointment times |  |  |  |  |  |  |  |
| **16** | A payment plan offered by the vet clinic |  |  |  |  |  |  |  |

ASK QD2a IF CODE 9>1 IN QD1

QD2a. Base = respondents indicating online information is influential in vaccination

Which of the following would be your **most preferred** online sources of information to learn how vaccines work / can help your cat(s)? *Select up to 2 responses.*

PGR: RANDOMIZE LIST, BUT KEEP ‘OTHER, SPECIFY’ LAST

| 1 | Veterinary clinic website page |
| --- | --- |
| 2 | Social media groups/posts (i.e., Facebook, Instagram) |
| 3 | Online videos (i.e., YouTube, TikTok) |
| 4 | Emails from the clinic |
| 5 | Pet blogs |
| 6 | Cat vaccine manufacturer website page |
| 7 | Other website pages [ANCHOR SECOND TO LAST] |
| 9 | Other, please specify:___________ [ANCHOR] |

ASK QD2b IF CODE 11>1 IN QD1

QD2b. Base = respondents indicating reminders from the clinic are influential in vaccination

Which of the following would be your **most preferred** way of receiving reminders from the vet clinic to schedule your cat’s vaccination appointment? *Select one.*

PGR: RANDOMIZE LIST BUT KEEP ‘OTHER, SPECIFY’ LAST

| 1 | Phone call reminder |
| --- | --- |
| 2 | Text message reminder |
| 3 | Postcard reminder |
| 4 | Email reminder |
| 9 | Other, please specify:___________ [ANCHOR] |

QD3. When thinking about vaccines for your cat(s), how familiar are you with each of the following?

**[RANDOMIZE LIST]**

|  |  | **Never heard of it**  **1** | **Somewhat familiar**  2 | **Very familiar**  **3** |
| --- | --- | --- | --- | --- |
| **1** | Non-adjuvanted vaccines |  |  |  |
| **2** | Low-volume / low dose vaccines *(i.e., 0.5ml instead of 1ml)* |  |  |  |

QD4. When thinking about vaccines for your cat(s), how important to you are each of the following?

**[RANDOMIZE LIST]**

|  |  | Not important  at all  1 | 2 | 3 | 4 | Extremely important  5 |
| --- | --- | --- | --- | --- | --- | --- |
| **1** | Lasts more than 1 year / longer duration of protection |  |  |  |  |  |
| **2** | Protects against as many diseases as possible in a single dose |  |  |  |  |  |
| **3** | Low risk of side effects |  |  |  |  |  |
| **4** | Is non-adjuvanted **[ONLY SHOW IF D3_1=2,3]** |  |  |  |  |  |
| **5** | Smaller injection volume *(i.e., 0.5ml instead of 1ml)* **[ONLY SHOW IF D3_2=2,3]** |  |  |  |  |  |
| **6** | Limited / fewer ingredients |  |  |  |  |  |
| **7** | Preservative free |  |  |  |  |  |
| **91** | Other, please specify **[ANCHOR LAST]** |  |  |  |  |  |

QD5a. When thinking about the length of protection offered by vaccines for your cat(s), assuming the following options were available which would you most prefer for **[SURVEYPET]**?

**[RANDOMIZE LIST]**

| **1** | A vaccine that offers 1 year of protection |
| --- | --- |
| **2** | A vaccine that offers 3 years of protection |
| **3** | No preference **[ANCHOR LAST]** |

**ASK QD5b IF QD5a=2 OR 3**

QD5b. Would you be willing to pay a premium for a vaccine for **[SURVEYPET]** that provides longer lasting protection (e.g., 3 years versus 1 year of coverage)?

| **1** | Yes |
| --- | --- |
| **2** | No |
| **3** | Maybe |

**ASK IF QA6=3 (VACCINATED CAT)**

QD6. Base = cat has been to veterinarian for vaccinations

If the vaccines **[SURVEYPET]** was given offered protection for 3 years, would you take **[SURVEYPET]** to the vet for wellness visits/checks in year(s) that he/she was not due for a repeat vaccine?

| **1** | Yes |
| --- | --- |
| **2** | No |
| **3** | Maybe |

**QD7.** Where do you **most commonly** seek out information / ask questions you have about vaccines for your cat(s)? *Select up to 5 responses.*

**[RANDOMIZE LIST]**

| **1** | My veterinarian |
| --- | --- |
| **2** | Pet Store |
| **3** | Friends / family |
| **4** | Online / internet pages |
| **5** | Social media groups (i.e., Facebook, Instagram) |
| **6** | Online videos (i.e., YouTube, TikTok) |
| **7** | Animal shelters |
| **8** | Breeder |
| **9** | Groomer |
| **10** | Veterinarian nurse/staff |
| **11** | Booklets/pamphlets about vaccines from the veterinary clinic |
| **12** | Other print materials (i.e., books, magazines, newspapers) |
| **13** | Vet clinic website |
| **91** | Other, please specify **[ANCHOR LAST]** |
| **99** | None; I do not seek out information for vaccines for my cat(s) **[ANCHOR LAST; EXCLUSIVE]** |

**SECTION E: PET OWNER SEGMENTATION TYPING TOOL QUESTIONS**

**OBJECTIVE – Classify owners into segments – Q94a, Q41a, Q50a, Q51a, Q52a, Q56a**

Next, we would like to learn more about you and your pet’s habits.

**Q94a.** In relation to preventative medicine, in general, please look at the following statements and tell us to what extent you agree or disagree with each one.

|  | Strongly  disagree | Disagree | Somewhat  disagree | Neither agree  nor disagree | Somewhat  agree | Agree | Strongly  agree |
| --- | --- | --- | --- | --- | --- | --- | --- |
| **4.** Vaccination is only necessary for puppies or kittens: | **1** | **2** | **3** | **4** | **5** | **6** | **7** |
| **7.** Regular vaccination is an opportunity for a health check on my cat: | **1** | **2** | **3** | **4** | **5** | **6** | **7** |

**Q41a**. How are the cat’s teeth cleaned? Please select all that apply.

**[Randomize list – multi punch]**

| Using dental treat / dental stick | **1** |
| --- | --- |
| They are cleaned at home | **2** |
| They are cleaned at the vets | **3** |
| Not cleaned **[EXCLUSIVE]** | **4** |

**Q50a**. Thinking about when you travel, how is the cat looked after? Please select all that apply.

**[Randomize list – multi punch]**

| The cat is boarded at kennel / cattery | **1** |
| --- | --- |
| The cat is boarded at the vet | **2** |
| The cat stays with a friend (or extended family) | **3** |
| The cat stays at home and is looked after there | **4** |
| The cat comes with me | **5** |
| Often times someone will stay behind to take care of the cat | **6** |
| The cat is never left alone **[SHOULD NOT BE EXCLUSIVE]** | **7** |

**Q51a**. How would you describe the cat’s role in your household? Please select all that apply.

**[Randomize list – multi punch]**

| Pest / vermin control | **1** |
| --- | --- |
| Guard | **2** |
| Emotional support / Therapy | **3** |
| Service animal | **4** |
| Hunting | **5** |
| Show / Competition | **6** |
| Breeding | **7** |
| Companion | **8** |
| Guide | **9** |
| Playmate | **10** |
| Working / farm pet | **11** |
| Like a child / fur baby | **12** |

**Q52a**. What is the main type of food you give to the cat? Please select all that apply.

**[Randomize list – multi punch]**

| Specialist or prescription diet from the vet (please specify) | **1** |
| --- | --- |
| Brand name (please specify) | **2** |
| Store brand (e.g. supermarket own brand) | **3** |
| Homemade (e.g. home cooked food specially for cats) | **4** |
| Human, or table, food, prepared for the cat | **5** |
| Leftover food / scraps | **6** |

**Q56a**. Which areas of your home, if any, is the cat not allowed to be in? Please select all that apply. **[Randomize list – multi punch]**

| Family bedroom(s) | **1** |
| --- | --- |
| Spare bedroom(s) | **2** |
| Dining room | **3** |
| Living room | **4** |
| Bathroom | **5** |
| Kitchen | **6** |
| Other | **7** |
| The cat has full access to every area of my home **[EXCLUSIVE]** | **8** |
| The cat is not allowed inside of my home **[EXCLUSIVE]** | **9** |

**SECTION F: Demographics**

**OBJECTIVE – Collect demographic information to use for profiling of results**

- **QE10/QE20/QE30/QE30a – household demos**
- **QE40/QE50/QE60/QE65/QE70 – cat specific demos**

Before we close, we just have a few more questions for classification purposes only.

QF10. Which of the following best describes the area in which you live?

**[RANDOMIZE LIST – SINGLE PUNCH]**

| Suburban | **1** |
| --- | --- |
| Urban | **2** |
| Rural | **3** |

QF15. What is your gender?

| Female | **1** |
| --- | --- |
| Male | **2** |
| Non-binary/third gender | **3** |
| Prefer to self-describe:_________________ | **4** |
| Prefer not to say | **5** |

QF20. Are you…?

| Single | **1** |
| --- | --- |
| Married or Domestic partnership | **2** |
| Widowed/Divorced | **3** |

QF30. How many people are living in your household?

| Number of people | ______ |
| --- | --- |

**[RANGE = 1 – 99]**

QF40. Please tell us again what **[IF QS6=1, SHOW:** is **/ IF QS6>1, SHOW:** are**]** the **[IF QS6=1, SHOW:** name **/ IF QS6>1, SHOW:** names**]** of your **[IF QS6=1, SHOW:** cat **/ IF QS6>1, SHOW:** cats**]**?

[MANDATORY TEXT BOX(ES). PROVIDE SAME NUMBER OF BOXES AS CATS IN QS6. FLAG IF RESPONSES DO NOT MATCH QA1.]

**CLOSE:** Thank you! Those are all the questions we have for you today.
